# Ventricular tachycardia: Focal pulsed field electroporation as a rescue therapy

**DOI:** 10.1016/j.hroo.2024.09.016

**Published:** 2024-11-20

**Authors:** Rita Reis Santos, Mariana Sousa Paiva, Rita Amador, Daniel Matos, Pedro Carmo, Pedro Adragão

**Affiliations:** Cardiology Department, Hospital de Santa Cruz, Centro Hospitalar de Lisboa Ocidental, Carnaxide, Portugal

**Keywords:** Ventricular tachycardia, Arrhythmia, Electroanatomic mapping, Ablation, Focal pulsed field electroporation

**Key Findings**

▪ Electroanatomic maps are essential for understanding and clarifying the substrate of each arrhythmia.
▪ Pulsed field ablation, as a novel ablation modality, seems to be a safe and efficient approach for recurrent ventricular tachycardia that is refractory to conventional therapies.
▪ Focal pulsed field ablation with an epicardial approach can penetrate the deep endocardial scar tissue, interrupting the ventricular tachycardia.

## Introduction

Ventricular tachycardia (VT) is a potentially fatal cardiac rhythm disturbance, caused by abnormal focal activity or, more commonly in patients with cardiomyopathy, by re-entrant circuits in the ventricular myocardium.^1^^,^^2^ Management of these patients typically involves the implantation of an implantable cardioverter-defibrillator (ICD) to prevent sudden cardiac death, along with therapy for the underlying heart disease.^2^^,^^3^ However, ICDs do not prevent VT, and a significant number of patients experience a high VT burden, highlighting the need for therapeutic strategies aimed at VT suppression. Pulsed field ablation (PFA) is a novel ablation modality that induces cell death through irreversible electroporation of cell membranes, specifically targeting cardiac myocytes and sparing structures such as nerves or blood vessels.^4^^,^^5^ In this case report, we describe a complex case of a male patient who presented with VT refractory to conventional therapy and was successfully treated with focal PFA.

## Case presentation

A 71-year-old man with a known history of ischemic cardiomyopathy requiring left anterior descending artery (LAD) percutaneous revascularization in 1986, reduced left ventricular ejection fraction, and an ICD implanted in 2008, presented with electrical storm and multiple ICD shocks. These were triggered by a sustained monomorphic VT with right bundle branch block morphology and right superior axis, followed by multiple episodes of antitachycardia pacing and shocks. The patient had previously undergone 2 endocardial catheter ablation procedures for VT (in 2018 and 2019), and an epicardial ablation in 2022, but experienced a relapse despite high doses of bisoprolol and amiodarone. Echocardiographic examination revealed a severely reduced ejection fraction of 30%, along with ventricular akinesia/hypokinesia corresponding to the LAD territory and an apical aneurism. Previous cardiac computed tomography angiography showed cicatricial thinning with fat metaplasia in the LAD territory, as well as a calcified intracardiac apical thrombus.

Given the presentation as an electrical storm, refractory to conventional therapy, 3 previous ablation procedures, and the presence of an intracardiac thrombus, a subxiphoid epicardial approach was planned.

**Figure 1:**
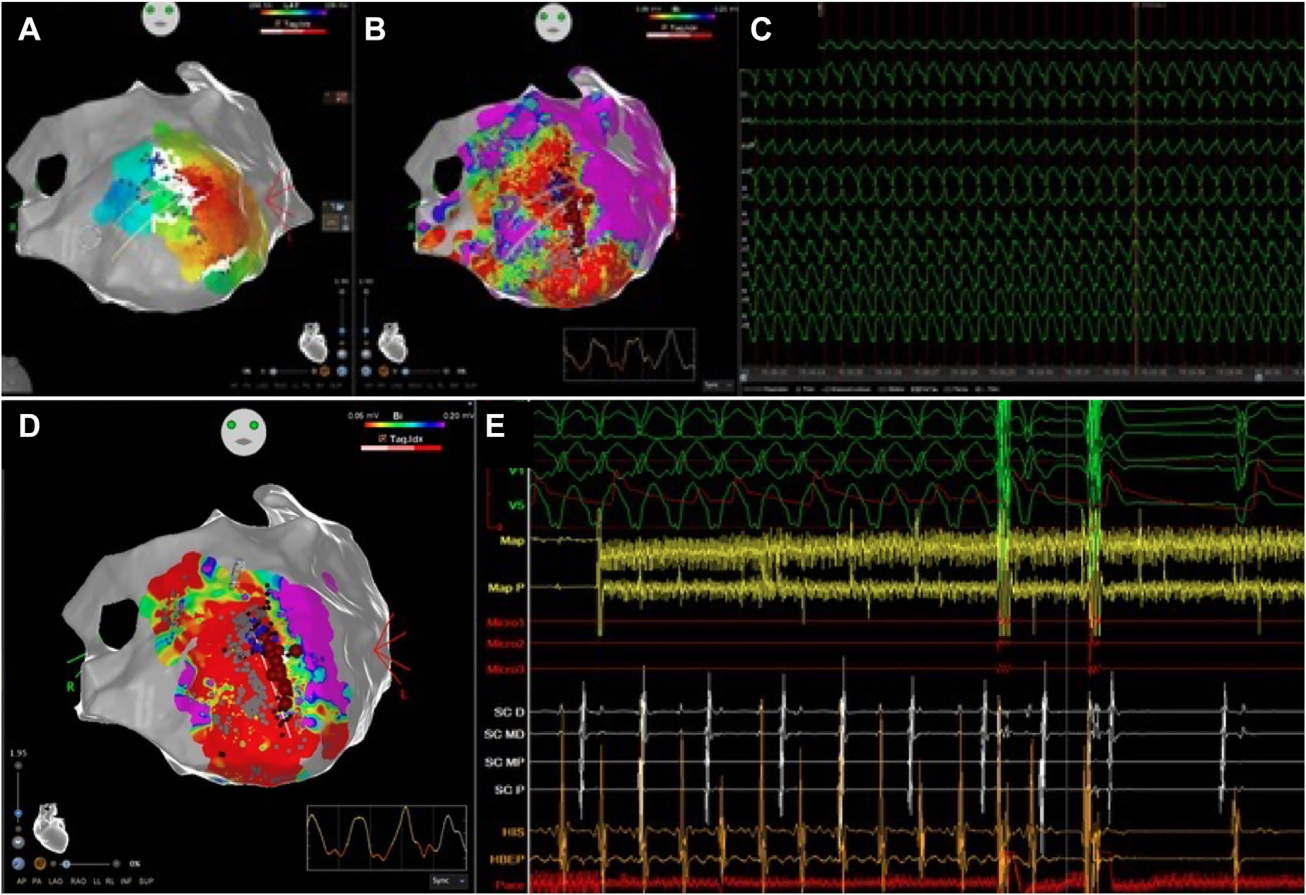
A: Electroanatomic maps of the left ventricle showing an anterior and apical scar. B: Substrate map of the left ventricle showing pre–ventricular tachycardia (VT) termination regions of the patchy apical scar. C: Electrogram from the VT map. D: Substrate map after VT termination. E: Electrogram showing pulsed field ablation and VT termination.

When the procedure started, the patient was in sinus rhythm, and endocardial mapping of the LV was performed using the CARTO version 7.2 PRIME electroanatomic mapping system (Biosense Webster) and a multipolar high-density catheter, showing an apical and anterior wall scar. Multiple areas of local abnormal ventricular activity were identified in this region (Figures 1A and 1B). VT was easily induced by programmed ventricular stimulation, presenting as a slow VT with right bundle branch block morphology (cycle length 480 ms). The activation map evidenced a probable exit point at the lateral edge of the scar (presystolic signals). The clinical VT was hemodynamically unstable, and 60 ms of the VT remained unmapped, suggesting the presence of a midmyocardial or epicardial portion of the circuit. Further mapping was not pursued due to the hemodynamic instability associated with the clinical VT. VT ablation was performed using a SmartTouch catheter (Biosense Webster), connected to a pulsed field energy generator (Galaxy Centauri; Galaxy Medical), enabling a focal contact force–guided ablation using electroporation. The system delivers biphasic, monopolar pulsed electric field energy at 3 selectable energy settings (19, 22, and 25 A) through the tip electrode of the ablation catheter. We used 25 A—the maximum current. The pulses are synchronized with the QRS complex.

VT was terminated during ablation at the exit point and rendered noninducible (Figures 1D and 1E). After converting to sinus rhythm, we further eliminated all local abnormal ventricular activity in the epicardial scar. The success of the ablation was also confirmed by attempting reinduction with extensive ventricular stimulation, which resulted in no further VT induction. The total procedure time was 2 hours and 55 minutes.

Based on coronary computed tomography angiography findings of cicatricial thinning with fat metaplasia in the LAD territory, the area was deemed nonviable. Despite that, to prevent vasospasm, intravenous nitroglycerin was prophylactically administered before each application. No procedural complications occurred. The patient was discharged on the fourth day after the procedure, maintaining the same doses of bisoprolol and amiodarone. During the 3 following months, no recurrences were documented by the ICD.

## Discussion

VT is a potentially fatal cardiac rhythm disturbance, and the risk of VT and consequent sudden cardiac death depends on the underlying cardiac conditions, as well as on other medical and genetic predispositions.^1^^,^^2^

While ICDs do not prevent VT, patients may still experience shocks or antitachycardia pacing during arrhythmic events, mainly in the first year after implantation. This can lead to a decreased quality of life and an increased risk of heart failure hospitalizations and death, highlighting the need for effective therapeutic strategies to suppress VT. Additionally, according to the recent (Does Timing of VT Ablation Affect Prognosis in Patients With an Implantable Cardioverter-defibrillator?) PARTITA trial, early referral for VT ablation in patients with ischemic or nonischemic cardiomyopathy, as soon as the first ICD shock occurs, reduces the risk of death and worsening heart failure hospitalization.^6^

PFA is a novel ablation modality that induces cell death through irreversible damage to cell membranes. It can be specifically tailored to target cardiac myocytes while sparing structures such as nerves or blood vessels. There is now a considerable body of evidence supporting its use for pulmonary vein isolation, and some recent reports also support PFA’s use in VT ablation due to its ability to create deep and homogeneous lesions through scar tissue.

This approach reduces the risk to surrounding structures, minimizes scar contraction, and decreases the risk of coagulum formation.^4^^,^^5^ Although we were unable to map all the tachycardia cycle length in the epicardium, the lack of VT inducibility at the end of the procedure suggests that either the focal PFA lesions penetrated deeply enough to reach the endocardium or that the critical isthmus was in the epicardial region. We believe that our approach was a safe and effective solution for this patient. We decided to perform PFA because previous radiofrequency ablations had not been effective, and considering the patient’s ischemic condition and the evidence of fat metaplasia in the LAD territory, we believed that PFA’s greater ability to create deeper lesions in heterogeneous scarred myocardium would be a suitable option for this patient.

## Conclusion

Focal PFA is a new ablation strategy that can potentially improve VT ablation outcomes due to its ability to better penetrate the scarred myocardium. In our case, focal PFA proved to be a safe and effective approach for an ischemic patient with VT refractory to conventional therapy and catheter RF ablation.

## Disclosures

The authors have no conflicts to disclose.
